# Exosomal let-7b-5p derived from Aspergillus fumigatus-treated human corneal epithelial cells promotes M1 macrophage activation via targeting SOCS-1

**DOI:** 10.3389/fimmu.2025.1548802

**Published:** 2025-06-04

**Authors:** Xiaoming Yu, Xinyi Wu

**Affiliations:** ^1^ Department of Ophthalmology, Qilu Hospital of Shandong University, No.107, Wenhua Xilu, Jinan, Shandong, China; ^2^ Department of Ophthalmology, Shandong Provincial Third Hospital, Shandong University, Jinan, Shandong, China

**Keywords:** fungal keratitis, exosomes, let-7b-5p, SOCS-1, M1 macrophage activation

## Abstract

**Purpose:**

This study aimed to explore the impact of exosomal miRNAs derived from *Aspergillus fumigatus* (*A. fumigatus*)-treated human corneal epithelial cells (HCECs) on M1 macrophage activation. We further clarified the mechanisms contributing to M1 macrophage activation in fungal keratitis.

**Methods:**

Exosomes were harvested from *A. fumigatus*-treated HCECs. Transmission electron microscopy, particle size analysis, and western blotting were performed to identify exosomes from HCECs. A laser confocal microscope was used to trace the exosomes. Macrophages were incubated with exosomes derived from *A. fumigatus*-treated HCECs. Global miRNA expression profiling of exosomes was assessed by high-throughput differential gene expression analysis. PCR and western blotting were used to detect the expression of M1-related proteins and SOCS-1. PCR was performed to detect the expression of pro-inflammatory cytokines and let-7b-5p. A dual-luciferase reporter assay was used to confirm the direct targeting of let-7b-5p.

**Results:**

*A. fumigatus*-treated HCEC-derived exosomes notably promoted M1 macrophage activation and the production of inflammatory cytokines. Let-7b-5p was overexpressed in exosomes. Let-7b-5p inhibitors suppressed the M1 immune response induced by exosomes. Overexpression of let-7b-5p repressed the expression of SOCS-1, whereas the let-7b-5p inhibitor dramatically increased the expression of SOCS-1. Moreover, a dual-luciferase reporter assay confirmed that SOCS-1 is a direct target of let-7b-5p.

**Conclusions:**

Let-7b-5p is secreted by *A. fumigatus*-treated HCECs and transferred to macrophages via exosome secretion. The communication between *A. fumigatus*-treated HCECs and macrophages was facilitated by exosomal let-7b-5p, resulting in the activation of M1 macrophages. The exosome/let-7b-5p/SOCS-1 axis is vital for innate immunity against fungal keratitis and provides insights into the molecular mechanisms involved in this condition.

## Introduction

Fungal keratitis (FK), an infectious disease that can cause severe visual impairment and even loss of vision, is widely distributed worldwide, with a particularly high prevalence in large agricultural countries and tropical regions (1). In patients with keratitis, a history of corneal trauma, chronic ocular surface disease, corneal surgery, corticosteroid medication, or contact lens use is considered a risk factor for fungal corneal infection. The treatment of FK is currently a major challenge due to the body’s poor response to antifungal medications and the reduced sensitivity of FK to antifungal medications compared to other types of infectious keratitis (2). *Aspergillus fumigatus* (*A. fumigatus*), a common causative agent of fungal infectious diseases, can parasitize a wide range of crops in nature. Corneal infections caused by *A. fumigatus* can lead to blindness in severe cases (3).

In response to different stimuli, macrophages play a crucial role in innate immunity and inflammation through expressing a diverse array of surface receptors and metabolic enzyme patterns (4–8). Macrophages typically have two different polarized states known as M1 and M2. The M1 state is activated in response to tissue damage, leading to an increase in pro-inflammatory cytokines such as inducible nitric oxide synthase (iNOS), phospho-p65, and interleukin-12 (IL-12). M2-polarized macrophages suppress inflammation by generating anti-inflammatory molecules like IL-10 and arginase-1, potentially facilitating tissue healing following inflammation (9–11).

Several studies have investigated the effects of therapeutic agents on FK. Yu et al. reported that indoleamine 2,3-dioxygenase (IDO) can regulate FK by affecting macrophage function through the CCL2/CCR2 pathway (12). Researchers have demonstrated that pseudolaric acid B (PAB) can ameliorate FK by altering macrophage polarization (13). Honokiol has antifungal and anti-inflammatory properties that influence macrophage polarization in *A. fumigatus* FK and can be developed as a potential and safe medication for FK (14). All of the above studies confirmed the important role of macrophages in FK caused by *A. fumigatus*. However, the mechanisms underlying macrophage activity in such processes require further investigation.

Extracellular vesicles, mainly exosomes released by all cell types, were originally thought to be carriers of cellular waste (15–17). Over the past few decades, multiple researchers have found that exosomes facilitate intercellular communication by transferring specific cargoes, such as lipids and genetic materials (18).

Recent studies have highlighted the role of exosomal miRNAs in macrophage polarization across different disease settings. For instance, exosomal miR-19b-3p derived from tubular epithelial cells promotes M1 macrophage activation through the SOCS1/NF-κB pathway, contributing to tubulointerstitial inflammation in kidney injury (19). Similarly, exosomal miR-374b-5p from tubular epithelial cells has been demonstrated to worsen renal ischemia/reperfusion injury by activating M1 macrophages, further reinforcing the role of exosomal miRNAs in inflammatory processes (20).

Another study found that extracellular vesicles from albumin-induced tubular epithelial cells promote M1 macrophage polarization by targeting Klotho, accelerating the progression of diabetic kidney disease (21). Among fungal infectious diseases, aspergillosis occurs frequently. In this study, we investigated a specific aspect of the immune response in A. fumigatus-related FK, focusing on the role of exosomal let-7b-5p in promoting M1 macrophage polarization through SOCS1 targeting. While these studies primarily focus on renal inflammation, little is known about the role of exosomal miRNAs in FK. Our study aims to bridge this gap by investigating whether exosomal let-7b-5p secreted by Aspergillus fumigatus-treated corneal epithelial cells contributes to M1 polarization through the SOCS1 pathway, a mechanism shared with kidney injury models.

Studies have also revealed that miRNAs are often found in exosomes and likely play essential roles in regulating cellular functions (22, 23). Zhao et al. revealed that miR-934, which is enriched in tumor-derived exosomes, is involved in the metastasis of colorectal cancer cells by promoting macrophage M2 polarization in the tumor microenvironment (24). As previously documented, immune cells can also release exosomal miRNAs and modulate inflammatory responses (25). In addition, numerous studies have confirmed that miRNAs play important roles in FK (26–28). However, it is not yet known whether exosome-derived miRNAs affect macrophage polarization during FK disease progression. With the above background in mind, this study was designed to analyze the differences in miRNA expression levels in *A. fumigatus*-treated HCECs and to detect the effect of exosome-derived miRNAs on macrophage polarization in FK.

## Materials and methods

### Aspergillus fumigatus strain

*A. fumigatus* (CCTCC 93024) was purchased from the Type Culture Collection Center of China and cultivated on Sabouraud dextrose agar (213400, BD, USA) in an incubator at 37 degrees Celsius for 7 days. Hyphal fragments and conidia were selected and subjected to Sabouraud liquid medium before being agitated at 25 degrees Celsius for 16 hours at 450 rpm. The *A. fumigatus* solution was rinsed three times with PBS, followed by heat inactivation of the hyphae at 56 degrees Celsius for 60 minutes. The mycelia were then fragmented into 20–40 micrometer pieces using a tissue grinder and utilized at a concentration of 1 × 10^6^ hyphal fragments per milliliter.

### Cell culture and treatment

The HCECs immortalized with SV40 and generously provided by Professor Fu-Shin Yu at Wayne State University were grown in a mixture of Dulbecco’s modified Eagle’s medium (DMEM/F12) from Gibco (Grand Island, NY, USA), along with 50% defined keratinocyte serum-free medium (KSFM) and 1% penicillin/streptomycin.

Human embryonic kidney cells (HEK-293T) were purchased from Procell, Wuhan, China. Cells were cultured using DMEM containing 10% FBS and 1% penicillin-streptomycin and maintained in a constant temperature incubator at 37°C with 5% CO_2_.

The THP-1 monocytic cell line was sourced from ATCC and cultured at 37°C in a humidified atmosphere with 5% CO_2_ using RPMI 1640 medium supplemented with 10% FBS, 100 U/mL penicillin G, and 100 µg/mL streptomycin sulfate. In order to induce the differentiation of THP-1 cells into macrophages, 4 × 10^5^ actively growing cells were treated with 100 ng/mL of PMA (Sigma) and then incubated for 48 hours. The resulting macrophage-like cells were harvested for further experiments.

### Isolation and identification of exosomes

HCECs were exposed to either *A. fumigatus* or exosome-free PBS in DMEM/F12 + KSFM medium for a duration of 12 hours. Following incubation, the culture supernatant of HCECs underwent a pre-clearance process through centrifugation at 2000 g, 4°C for 30 min, followed by centrifugation at 1 × 10^4^g, 4°C for 45 min. The supernatant was then filtered using a 0.22 µm membrane. Exosomes were separated from the supernatant through ultracentrifugation at 1.1 × 10^5^ g, 4°C for 70 min and subsequently washed in PBS using the same ultracentrifugation settings. The ultracentrifugation procedures were carried out utilizing the Hitachi CP100MX ultracentrifuge (HITACHI, Japan). The exosomes were visualized using a transmission electron microscope (TEM) HT7800 (HITACHI, Japan), and their diameter was determined using Nano-FCM N30E (NanoFCM, China). Western blot (WB) analysis was conducted to identify the presence of exosomal surface markers such as TSG101, CD9, and CD63, as well as the absence of calnexin, a negative marker for exosomes.

### Tracking of labeled exosomes

Exosomes obtained from HCECs treated with *A. fumigatus* or a PBS control were stained with PKH67 (ThermoFisher, USA) and then exposed to macrophages for 24 hours. After washing, the macrophages were stained with Calcein-AM (ThermoFisher, USA) for 20 minutes, and images were visualized using an inverted fluorescence microscope (Nikon, Japan).

### Treatment of macrophages

Macrophages were exposed to HCEC-derived exosomes at a concentration of 10 μg/mL in 50 μL PBS for a 24-hour incubation period. Additionally, exosomes obtained from inhibitor- or control-transfected HCECs, also at a concentration of 10 μg/mL in 50 μL PBS, were added to the macrophage culture medium and incubated for 24 hours. The cells were harvested for subsequent analysis. This concentration was selected based on preliminary optimization and prior literature. Particle number was not directly quantified using nanoparticle tracking or NanoFCM technologies. We recognize this as a limitation and plan to incorporate particle-based quantification in future studies for more precise standardization.

### miRNA expression profiles

Exosomes obtained from HCECs treated with *A. fumigatus* (n = 3) or PBS controls (n = 3) were lysed, and total RNA was extracted using the miRNeasy Serum/Plasma Kit (217184, QIAGEN, Germany) following the manufacturer’s instructions. Subsequently, libraries were prepared utilizing the QIAseq miRNA Library Kit (331505, QIAGEN, Germany) and subjected to miRNA profiling on the Illumina Xten platform (Illumina, USA) as per the manufacturer’s guidelines. The data analysis was conducted based on fold change and p-value.

### Cell transfection

To regulate the level of let-7b-5p, let-7b-5p inhibitors (5’-GGUAGUAGGUUGUGUGGUU-3’) and inhibitor controls (5’-CAGTACTTTTGTGTAGTACAA-3’), as well as let-7b-5p mimics (5’-TGAGGTAGTAGGTTGTGTGGTT-3’) and mimic controls (5’-UUCUGCCGAAUGUCACGUTT-3’), were procured from Sangon Biotech (Shanghai, China). The above-mentioned sequences were transfected into cells at 65% confluency using jetMESSENGER (101000056, Polyplus, France). Cells were harvested after 24 hours of culture at 37°C with 5% CO_2_ following transfection.

### Western blot

Proteins were extracted using RIPA lysis buffer (P0013B, Beyotime, China) supplemented with 1 mM PMSF (BL1426A, Labgic, China) as a protease inhibitor. The samples were electrophoretically separated on 10% SDS-PAGE gels using a Rapid Gel Preparation Kit (PG113, Epizyme, China) and transferred to PVDF membranes (IPVH00010, Merck, USA) at a continuous current of 200 mA for 80 minutes. Subsequently, the samples were incubated overnight on a shaking table at 4°C with primary antibodies against CD9 (ab307085, Abcam, USA), CD63 (A19023, Abclonal, China), TSG101 (ab83, Abcam, USA), Calnexin (A24433, Abclonal, China), p65 (A19653, Abclonal, China), p-p65 (AP0123, Abclonal, China), SOCS1 (ab280895, Abcam, USA), GAPDH (AC001, Abclonal, China), and iNOS (A3774, Abclonal, China). The membranes were then washed three times for 10 minutes each with TBST (T1081, Solarbio, Beijing), followed by incubation with HRP-conjugated anti-rabbit secondary antibody for 1 hour at room temperature. The PVDF membranes were then visualized using the ECL chemiluminescent kit and quantified using ImageJ software.

### RNA isolation and detection of mRNA and miRNA

Total RNA was extracted from both cells and exosomes using TRIzol. Complementary DNA (cDNA) was synthesized for total RNA and miRNAs using a reverse transcription kit (Takara, Shiga, Japan) and a First-Strand cDNA Synthesis Kit (tailing method) (Tsingke, China), respectively. Expression levels of mRNA were quantified using 1.1× EasyQ SYBR qPCR Mix (Tsingke, China) on a qPCR machine (Bioer, China).

Real-time analysis of mRNA levels was conducted using quantitative relative expression levels of target genes normalized to GAPDH and U6, respectively. The following table (**Table 1**) shows the primer sequences that were used.

**Table 1:** The primer sequences (forward and reverse) that used in the study.

Gene	Primer (5’-3’)
TNF-α	S: CCTCTCTCTAATCAGCCCTCTG
	AS: GAGGACCTGGGAGTAGATGAG
IL-6	S: ACTCACCTCTTCAGAACGAATTG
	AS: CCATCTTTGGAAGGTTCAGGTTG
let-7a-5p	S: GAACGTCGAAAAGAAAAGTCTCG
	AS: CCTTATCAAGATGCGAACTCACA
GAPDH	S: CCACTCCTCCACCTTTGAC
	AS: ACCCTGTTGCTGTAGCCA
U6	S: GCTTCGGCAGCACATATACTAAAAT
	AS: CGCTTCACGAATTTGCGTGTCAT
let-7b-5p	S: GCGCTGAGGTAGTAGGTT
	AS: GTGCAGGGTCCGA
let-7d-5p	S: AGAGGUAGUAGGUUGCAUAGUU
	AS: CUAUGCAACCUACUACCUCUUU
miR-30b-5p	S: GCAGTGTAAACATCCTACACTCA
	AS: ACCTAAGCCAGGGAGGTTA
miR-149-5p	S: CTCTGGCTCCGTGTCTTCAC
	AS: CTGCCCCAGCACAGCC
miR-30c-5p	S: AGCGTCGTATCCAGTGCAAT
	AS: GTCGTATCCAGTGCGTGTCG
MCP-1	S: CAGCCAGATGCAATCAATGCC
	AS: TGGAATCCTGAACCCACTTCT
iNOS	S: TTCAGTATCACAACCTCAGCAAG
	AS: TGGACCTGCAAGTTAAAATCCC
SOCS1	S: TTTTCGCCCTTAGCGTGAAGA
	AS: GAGGCAGTCGAAGCTCTCG

### Luciferase reporter assay

TargetScan (https://www.targetscan.org/vert_80/) was used to predict the binding sites of let-7b-5p on SOCS1. The 3’-UTR of SOCS1, along with the mutant SOCS1 3’-UTR sequence, was cloned into a pGL-U6-puromycin vector (Tsingke, China) for luciferase activity analysis. Next, cells were co-transfected with wild-type pGL3-SOCS1-3’UTR or mutant pGL3-SOCS1-3’UTR, along with a mimic control and let-7b-5p mimic, using jetMESSENGER transfection reagent as per the manufacturer’s instructions (Polyplus, France). Luciferase activity was measured using a reporter system 24 hours post-transfection (Promega, USA).

### Statistical analysis

All experiments were performed in triplicate, and the average ± SD (standard deviation) was used for statistical analysis. The student t-test was used for two cohorts. A multiple-comparison **test**, followed by Tukey’s *post hoc* test, was used for multiple groups. A P-value < 0.05 was considered statistically significant.

* All experiments were repeated independently at least twice, with each experiment containing three technical replicates. Data are presented as the mean ± standard deviation (SD). Statistical significance was evaluated using Student’s t-test for two-group comparisons and one-way ANOVA with Tukey’s *post hoc* test for multiple group comparisons. A p-value < 0.05 was considered statistically significant.

## Results

### Isolation and characterization of HCEC-derived exosomes

**Figure 1:**
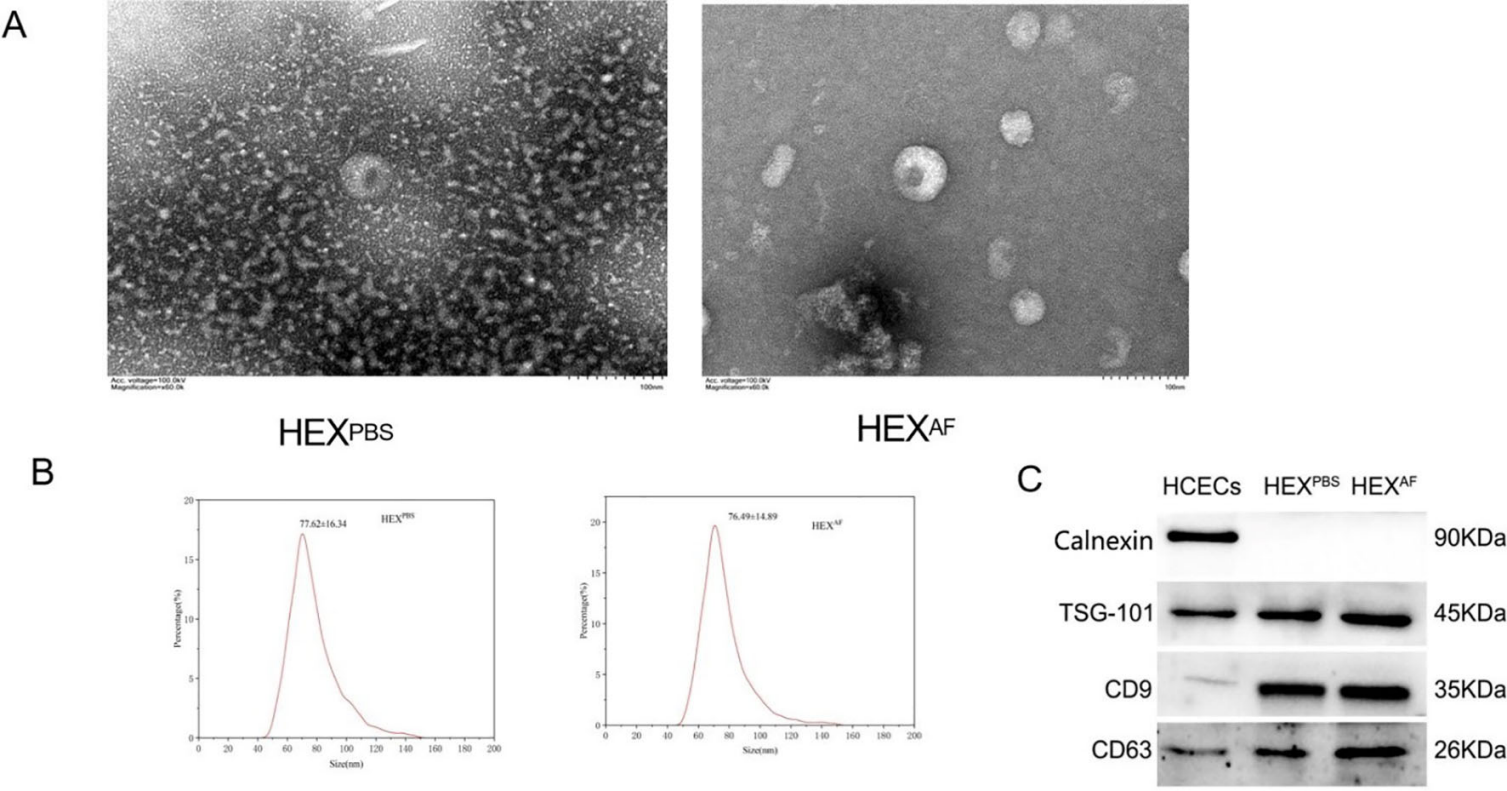
Identification of exosomes. **(A)** Detection of exosome morphology by transmission electron microscopy (200×); **(B)** Nanoflow technology to detect exosome size; **(C)** Exosome marker identification. HEX^PBS^, exosomes secreted by PBS-treated HCECs, HEX^AF^, exosomes secreted by *A*. *fumigatus*-treated HCECs.

HCECs were exposed to *A. fumigatus* or PBS for a period of 12 hours, after which exosomes were isolated from the culture supernatant. Exosomes were characterized as a biconcave discoid shape with a bilayer membrane structure, with respective diameters of 76.49 ± 14.89 nm and 77.62 ± 16.34 nm (**Figures 1A, B**). For both PBS-treated and *A. fumigatus*-treated HCECs, western blot analysis was performed to confirm whether exosomes were isolated. Thus, the presence of exosomal surface markers (CD9, CD63, and TSG-101), as well as the absence of Calnexin, the endoplasmic reticulum marker that is absent in exosomes and is used as a negative control, were assessed. While Calnexin was specifically absent in the exosomes, it was present in the HCECs themselves (**Figure 1C**). These findings confirm the successful isolation of exosomes.

### *A. fumigatus*-treated HCEC-derived exosomes promote M1 macrophage activation

**Figure 2:**
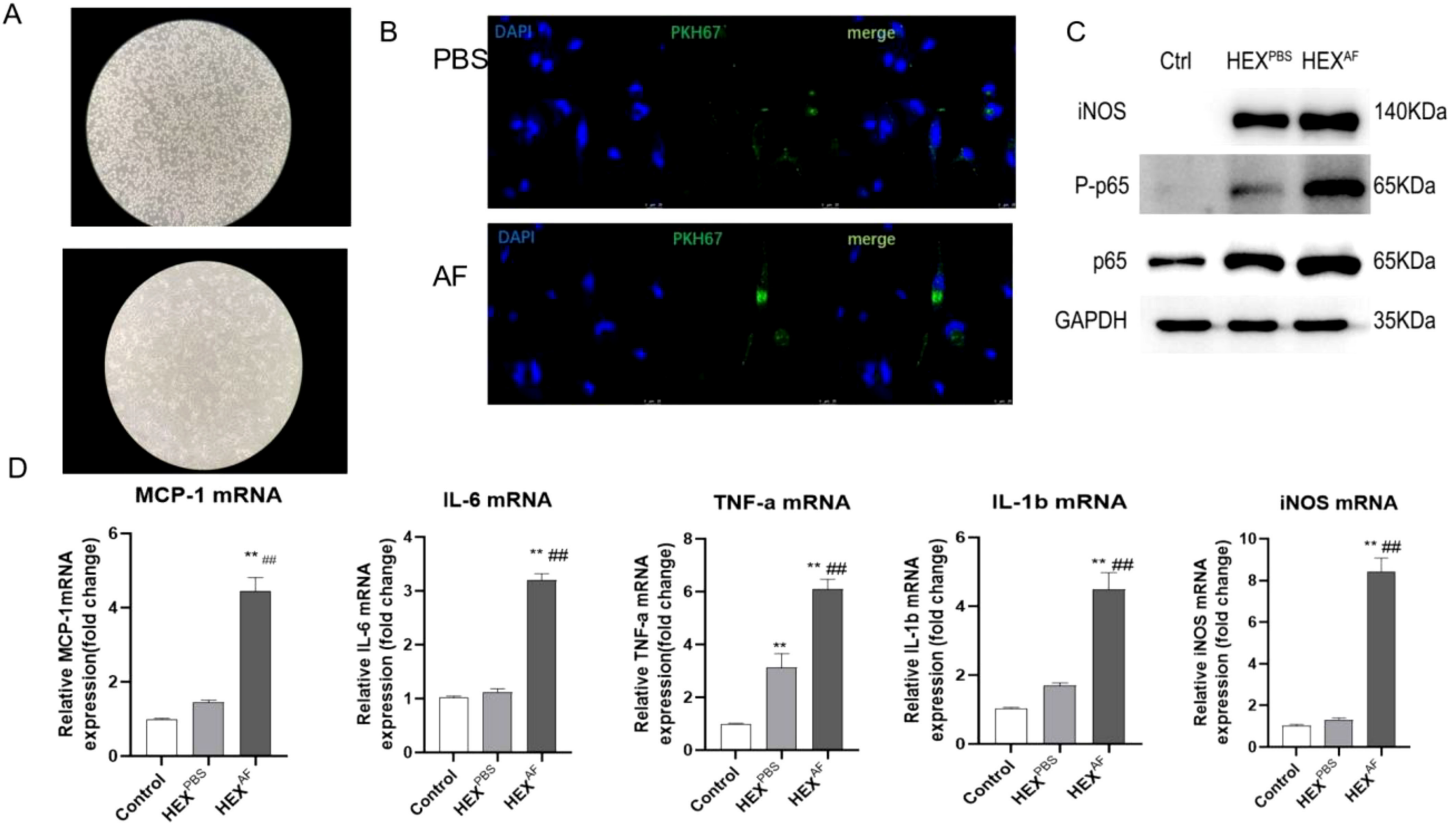
Role of exosomes secreted by *A*. *fumigatus*-stimulated HCECs for macrophages. **(A)** Induction of THP-1 cell differentiation into macrophages using PMA; **(B)** Macrophage uptake capacity for exosomes; **(C)** Protein expression levels of iNOS, phosphorylated p65 and Total p65; **(D)** The mRNA level of MCP-1 IL-6, TNF-a, IL-1β and iNOS; (** indicates P<0.01vs with control., ## indicates P<0.01 vs with HEX^PBS^) Data are exhibit as average ± SD of triple single experiments.

To explore the effect of exosomes derived from *A. fumigatus*-treated HCECs on macrophage activation, the macrophages were incubated with 10 µg/mL of harvested exosomes for 24 h. First, we induced THP-1 cells to differentiate into macrophages using 100 ng/mL PMA stimulation, and THP-1 cells were seen to adhere to the surface and differentiate into pseudopods after 48 hours of PMA induction (**Figure 2A**). The exosomes were labeled with the PKH67 staining solution and then incubated with macrophages for 24 h. Fluorescence tracking showed that the exosomes from two different sources could be taken up by macrophages and internalized into the cells (**Figure 2B**).

A western blot assay showed that the exosomes secreted by *A. fumigatus*-treated HCECs could promote iNOS expression and p65 phosphorylation levels (**Figure 2C**). In addition, RT-qPCR results showed that exosomes secreted by *A. fumigatus*-treated HCECs were able to promote the levels of macrophage inflammatory factors MCP-1, IL-6, TNF-α, IL-1β, and iNOS (**Figure 2D**). These results demonstrated that exosomes secreted by A. fumigatus-treated HCECs were able to induce M1-type macrophage polarization.

### The miRNA expression profile of exosomes derived from *A. fumigatus*-treated HCECs

**Figure 3:**
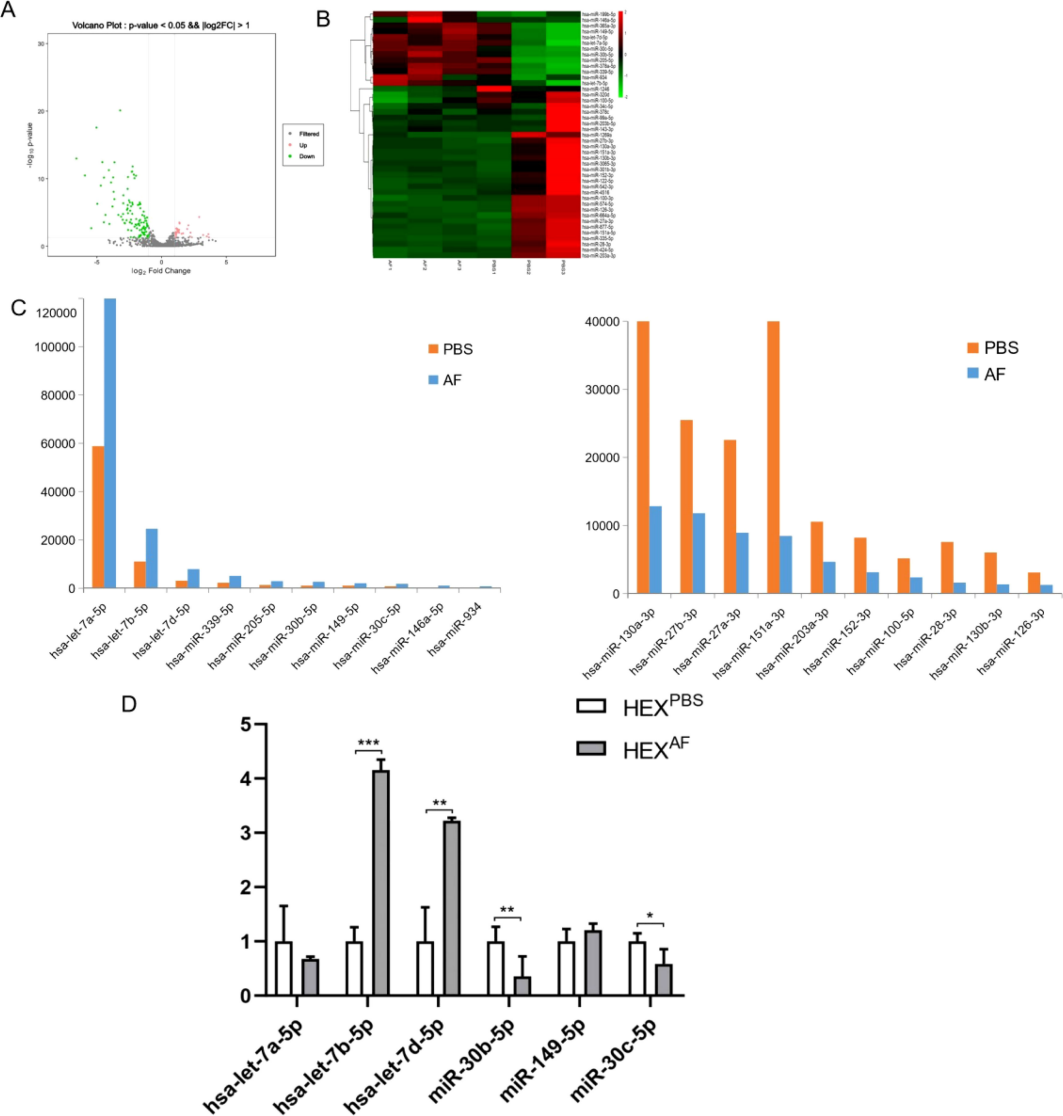
Transcriptome sequencing analysis and validation. **(A)** The volcano plot displays differentially expressed miRNAs in exosomes derived from **(A)** fumigatus-infected HCECs. Red dots represent significantly upregulated miRNAs, and blue dots indicate significantly downregulated miRNAs. Key miRNAs linked to immune modulation (e.g., let-7b-5p) and inflammatory pathways (e.g., miR-19b-3p, miR-374b-5p) are annotated for better interpretation.; **(B)** The heatmap illustrates the clustering of differentially expressed miRNAs in exosomes from infected vs. control HCECs. Functional annotations are provided for miRNAs associated with macrophage polarization, NF-κB signaling, and cytokine regulation, helping to contextualize their role in fungal keratitis pathogenesis.; **(C)** Top 10 miRNA expression levels obtained by sequencing; **(D)** RT-qPCR was used to verify the expression levels of let-7a-5p, let-7b-5p, let-7d-5p, miR-30b-5p, miR-149-5p, and miR-30c-5p in exosomes from different treatments; * Indicates P<0.05, ** indicates P<0.01 and *** indicates P<0.001. Data are exhibit as average ± SD of triple single experiments.

To explore the miRNA profiles of *A. fumigatus*-treated HCEC exosomes, the miRNA expression profiles were assessed using transcriptome sequencing. Hierarchical clustering analysis of miRNA expression detected that out of 1466 miRNAs, 141 (27 upregulated and 114 downregulated) were differentially expressed. After obtaining the results of transcriptome sequencing, a volcano plot of the number of differentially expressed miRNAs in two different groups of exosomes was obtained (**Figure 3A**), and the heat map of differential miRNAs was analyzed and obtained (**Figure 3B**). The volcano plot (**Figure 3A**) illustrates the distribution of differentially expressed miRNAs, highlighting key upregulated and downregulated miRNAs involved in macrophage polarization and immune regulation. Notably, let-7b-5p, miR-19b-3p, and miR-374b-5p are among the significantly altered miRNAs, suggesting their potential roles in exosome-mediated immune modulation during fungal keratitis. The heatmap (**Figure 3B**) provides an overview of the expression patterns of differentially expressed miRNAs, clustering them based on their relative abundance. Key miRNAs associated with inflammatory signaling pathways, macrophage activation, and immune modulation are prominently represented, further emphasizing their relevance in the host immune response to A. fumigatus infection. Subsequently, the top ten differentially expressed miRNAs in the exosomes of the PBS-treated group and the exosomes of the *A. fumigatus*-treated group were identified (**Figure 3C**). Finally, six miRNAs (let-7a-5p, let-7b-5p, let-7d-5p, miR-30b-5p, miR-149-5p, and miR-30c-5p) were selected for validation. The RT-qPCR results indicated that the expression of let-7b-5p was elevated in the exosomes of the *A. fumigatus*-treated group and was consistent with the sequencing results, while the validation results of let-7d-5p, although the same as the sequencing results, showed that the expression difference was not as significant as that of let-7b-5p (**Figure 3D**). Based on the above results, let-7b-5p was selected for subsequent experiments.

### Overexpression of let-7b-5p in macrophages after treatment with exosomes secreted from *A. fumigatus*- and PBS-treated HCECs

**Figure 4:**
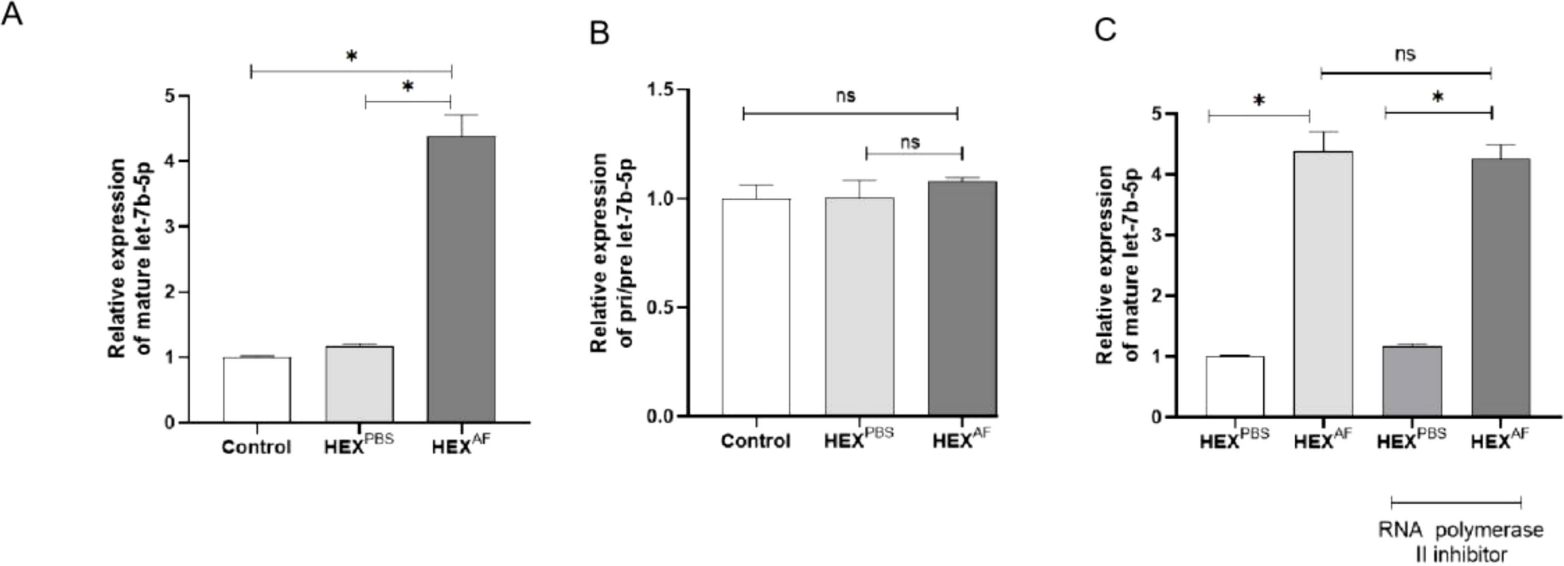
Level of let-7b-5p in exosome treated macrophage. **(A)** The gene level of mature let-7b-5p. **(B)** The level of Pri-/Pre-let-7b-5p. **(C)** Effect of RNA polymerase II inhibitor was detected by RT-qPCR. * Indicates P<0.05. Data are exhibit as average ± SD of triple single experiments. ns, not significant.

To confirm that *A. fumigatus*-treated HCEC-secreted let-7b-5p can be internalized into macrophages via exosomes, let-7b-5p levels were assessed in macrophages treated with exosomes separated from HCECs treated with *A. fumigatus* and PBS. An increased level of mature let-7b-5p (**Figure 4A**), but not pri-/pre-let-7b-5p (**Figure 4B**), was found in macrophages following treatment with exosomes from *A. fumigatus*-treated HCECs. In addition, the increase of let-7b-5p in macrophages exposed to exosomes from *A. fumigatus*-treated HCECs was not affected by an RNA polymerase II inhibitor (**Figure 4C**). These data suggested that *A. fumigatus*-treated HCEC exosomes containing let-7b-5p were internalized by macrophages.

### HCEC exosomes promote M1 macrophage activation via let-7b-5p

**Figure 5:**
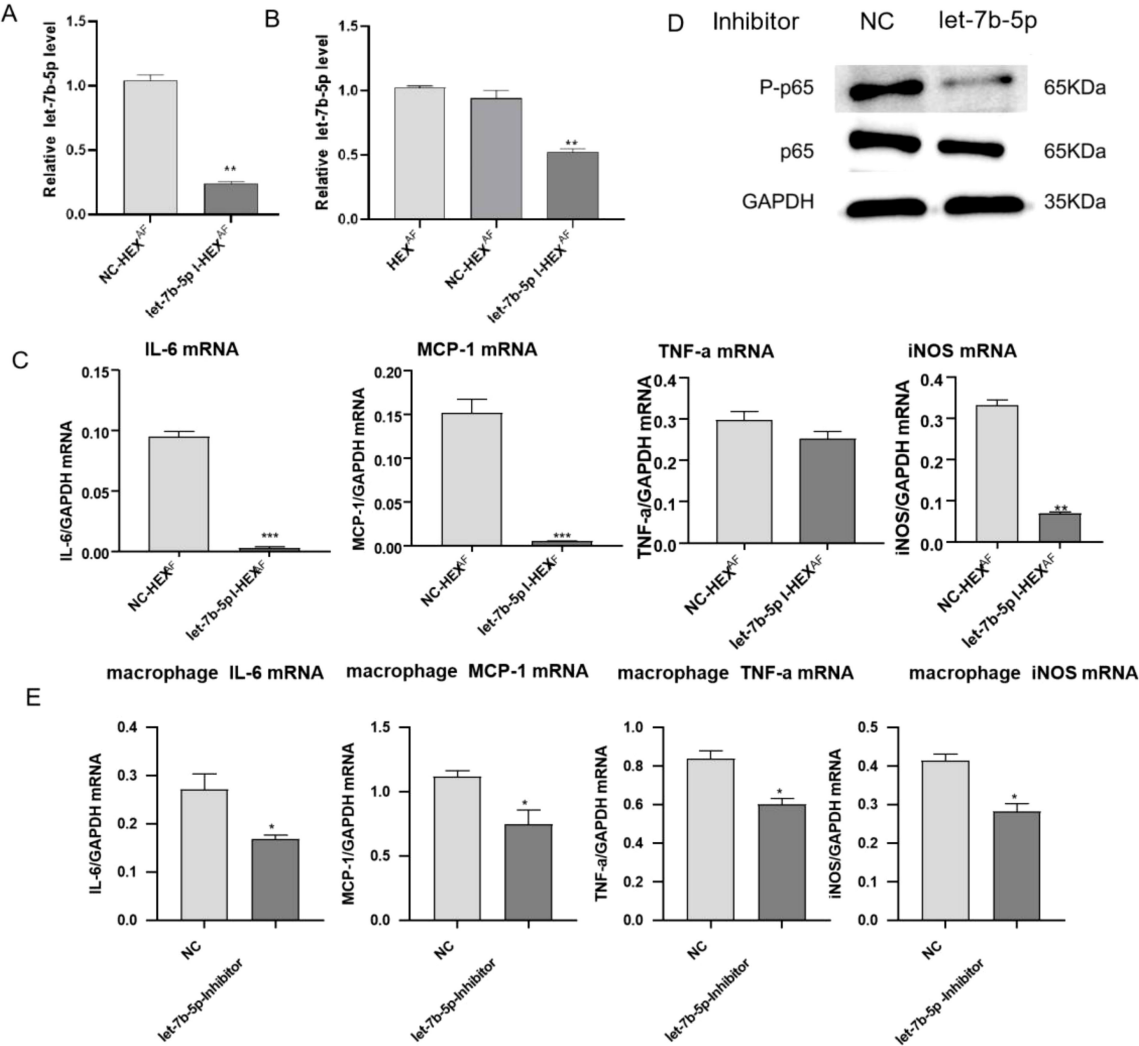
Effects of let-7b-5p in exosomes on macrophages. **(A)** The expression level of let-7b-5p in exosomes was measure by RT-qPCR. **(B)** RT-qPCR assay for let-7b-5p expression in macrophages. **(C)** The expression level of inflammation cytokines (IL-6, MCP-1, TNF-a and iNOS) in macrophages was measure by RT-qPCR. **(D)** The protein level of p-p65 and p65 in macrophages. **(E)** Detection of inflammatory cytokines in macrophages by RT-qPCR. * Indicates P<0.05, ** indicates P<0.01 and *** indicates P<0.001. Data are exhibit as average ± SD of triple single experiments.

To investigate the effect of let-7b-5p in exosomes secreted by HCECs on macrophage polarization, we transfected a let-7b-5p inhibitor into HCECs or macrophages. Then, the transfected HCECs were treated with *A. fumigatus*. We performed RT-qPCR to detect the expression of let-7b-5p in the exosomes from *modified A. fumigatus-treated HCECs*, * showing that let-7b-5p silencing significantly reduced the expression of let-7b-5p in the exosomes from these cells (**Figure 5A**). In addition, macrophages were incubated with exosomes from *A. fumigatus-treated transfected HCECs.* * The expression of let-7b-5p in the macrophages was detected by RT-qPCR. We found that exosomes from let-7b-5p-silenced HCECs led to a decrease in the expression of let-7b-5p in the macrophages (**Figure 5B**). Consistently, inhibition of let-7b-5p significantly altered the expression levels of MCP-1, IL-6, and iNOS mRNA in macrophages treated with HCEC exosomes (**Figure 5C**). Moreover, macrophages transfected with the let-7b-5p inhibitor showed inactivation of NF-κB p65 phosphorylation and reversed the increase of inflammatory cytokines, including MCP-1, IL-6, iNOS, and TNF-α (**Figures 5D, E**), demonstrating that HCEC exosomes promote M1 macrophage activation via let-7b-5p.

### Exosomal let-7b-5p promotes M1 macrophage activation by targeting SOCS-1

**Figure 6:**
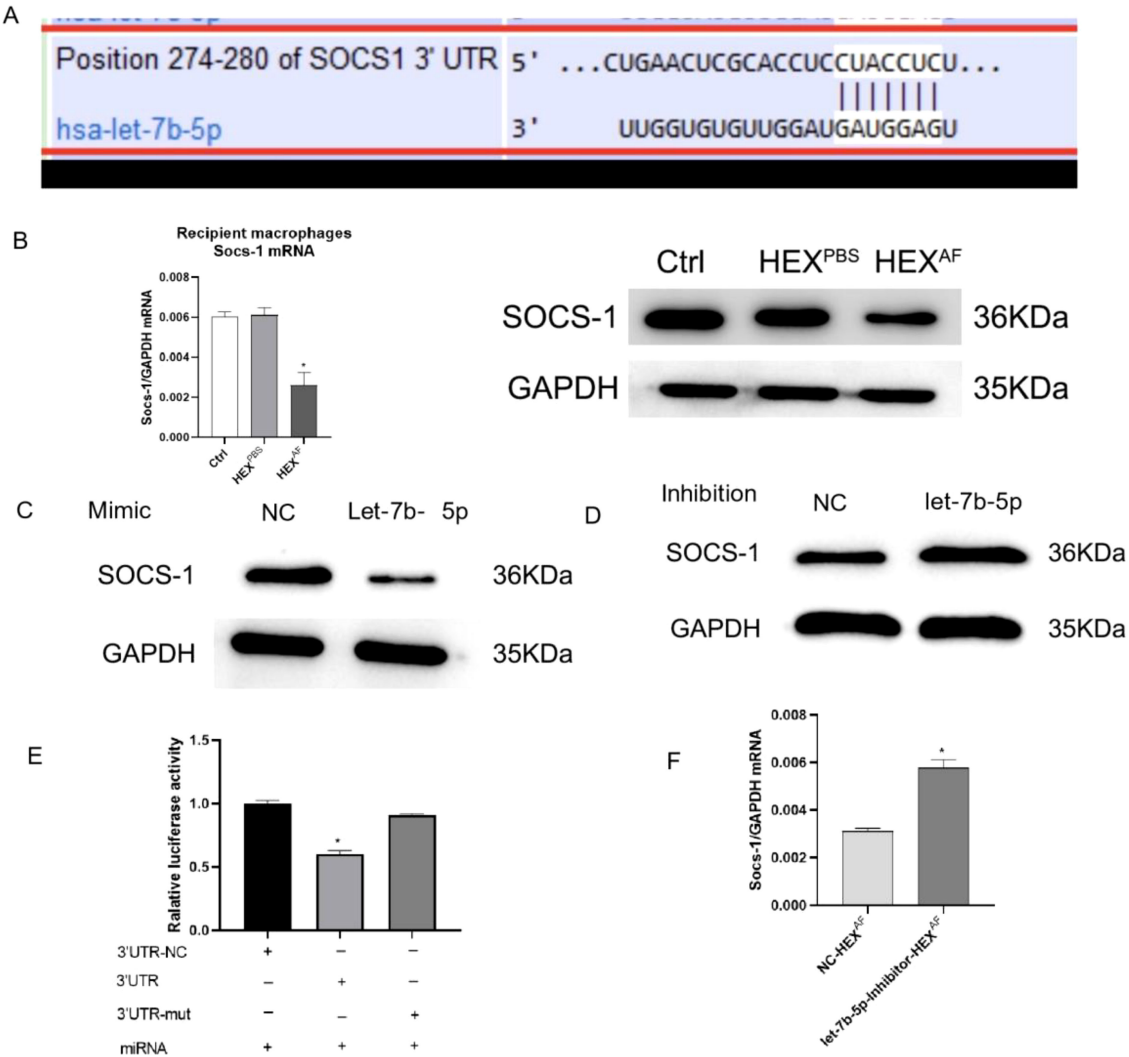
SOCS-1 is a downstream target gene of let-7b-5p. **(A)** The foreknowledge binding points between let-7b-5p and SOCS-1 was forecasted by Targetscan. **(B)** The protein and mRNA level of SOCS-1 in exosomes treated macrophages. **(C)** The protein level of SOCS-1 in let-7b-5p mimic transfected macrophages. **(D)** The protein level of SOCS-1 in let-7b-5p inhibitor transfected macrophages. **(E)** The dual relative luciferase assay was used for make sure the linkage between let-7b-5p and SOCS-1. **(F)** The mRNA level of SOCS-1. * Indicates P<0.05. Data are exhibit as average ± SD of triple single experiments.

Subsequently, our focus shifted towards elucidating the mechanism by which let-7b-5p facilitates M1 macrophage activation. According to the bioinformatics website (https://www.targetscan.org/vert_80/), suppressor of cytokine signaling 1 (SOCS-1) was identified as the potential target gene for let-7b-5p (**Figure 6A**). To investigate whether exosomal let-7b-5p targets SOCS-1 in recipient macrophages, exosomes enriched with let-7b-5p from *A. fumigatus*-treated HCECs were introduced to macrophages. The outcomes revealed a simultaneous downregulation of both mRNA and protein expression of SOCS-1 in the recipient macrophages (**Figure 6B**). Overexpression of let-7b-5p led to the suppression of SOCS-1 expression, while the let-7b-5p inhibitor notably upregulated SOCS-1 expression (**Figures 6C, D**). Subsequently, a dual luciferase reporter assay demonstrated a marked decrease in luciferase reporter activity upon let-7b-5p overexpression, while the activity of the SOCS-1 3’UTR-mutant luciferase reporter remained unaffected. This confirmed that SOCS-1 was a direct target gene of let-7b-5p (**Figure 6E**). Moreover, the depletion of SOCS-1 was observed in macrophages exposed to exosomes from HCEC cells transfected with the let-7b-5p inhibitor (**Figure 6F**), indicating that HCEC-derived exosomal let-7b-5p could potentially target SOCS-1 in macrophages.

**Figure 7:**
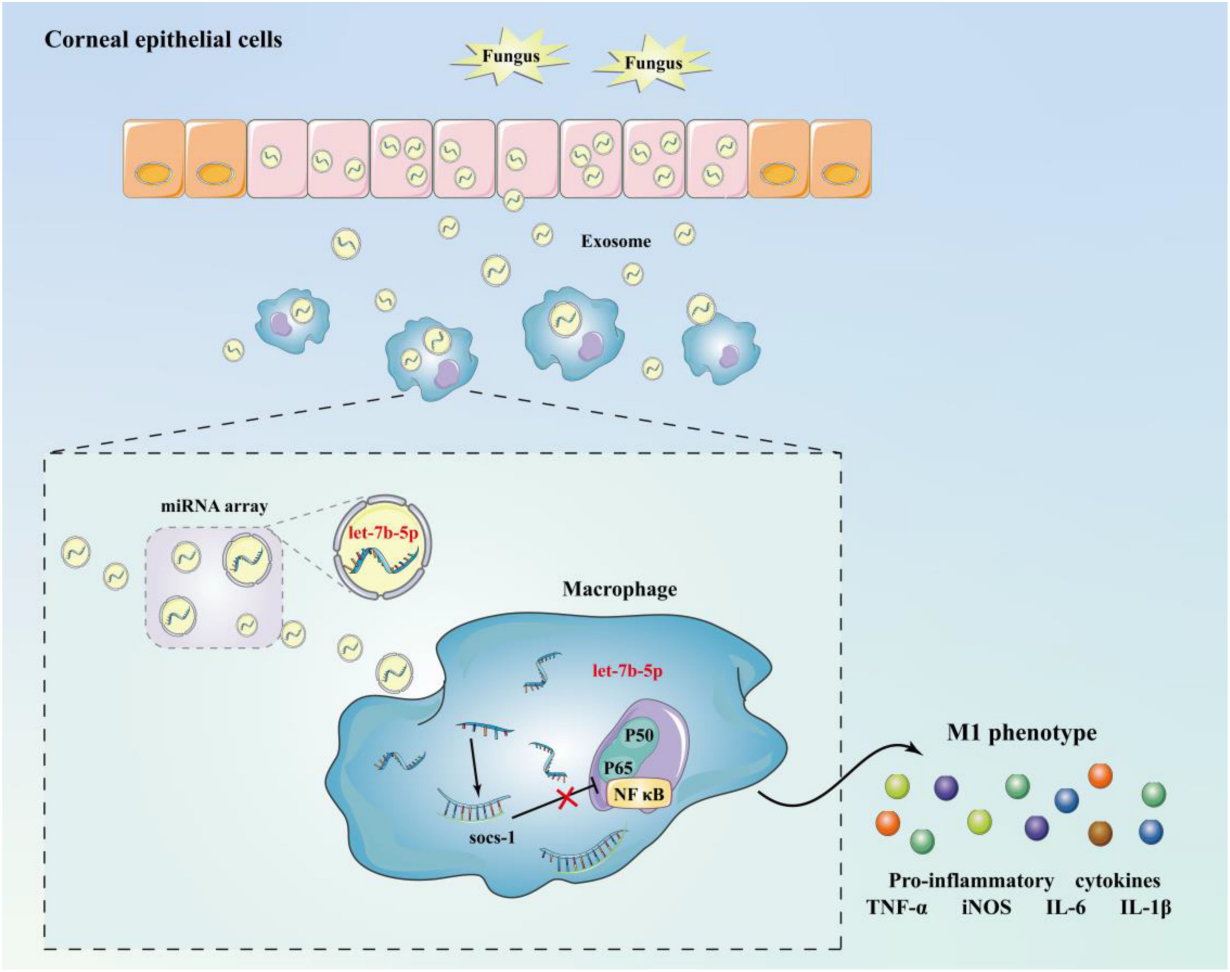
Schematic diagram of mechanism. Corneal epithelial cells secrete exosomes containing let-7b-5p during fungal infection and are internalised by macrophages, while let-7b-5p promotes M1 macrophage activation by directly targeting SOCS-1.

Taking all these results together, we conclude that corneal epithelial cells secrete exosomes containing let-7b-5p, which are internalized by macrophages during FK. Moreover, let-7b-5p promotes M1 macrophage activation by directly targeting SOCS-1. Communication between HCECs and macrophages via exosomal let-7b-5p enhances the initial inflammatory signaling of HCECs and promotes corneal antifungal natural immunity (**Figure 7**).

**Figure 8:**
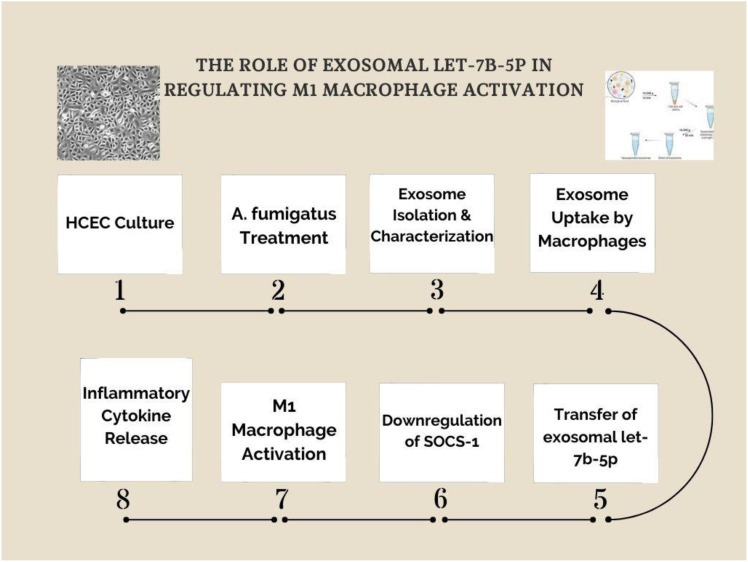
Exosomal let-7b-5p mediates M1 macrophage activation via SOCS-1 suppression. A schematic representation of the experimental workflow investigating the role of exosomal let-7b-5p in M1 macrophage activation. (1) Human corneal epithelial cells (HCECs) were cultured and (2) treated with *Aspergillus fumigatus* to induce exosome production. (3) Exosomes were isolated and characterized before (4) being taken up by macrophages. (5) The transfer of exosomal let-7b-5p to macrophages led to (6) the downregulation of SOCS-1, a key negative regulator of inflammatory signaling. (7) This resulted in M1 macrophage activation, characterized by (8) the release of inflammatory cytokines. The study highlights the exosome/let-7b-5p/SOCS-1 axis as a crucial regulatory pathway in fungal keratitis-related macrophage activation.

We developed a schematic representation of the experimental workflow to further illustrate the mechanistic pathway of exosomal let-7b-5p in M1 macrophage activation (**Figure 8**). The process begins with human corneal epithelial cells (HCECs) culture, followed by Aspergillus fumigatus treatment to induce exosome production. The isolated exosomes are then taken up by macrophages, where the transfer of let-7b-5p results in SOCS-1 suppression. This leads to the activation of M1 macrophages and an increase in inflammatory cytokine secretion. This pathway highlights the exosome/let-7b-5p/SOCS-1 axis as a key modulator of macrophage polarization in fungal keratitis.

## Discussion

Fungal keratitis is a serious blinding eye disease caused by infection with pathogenic fungi. Immune tolerance and resistance are important immune defense mechanisms, and the dynamic balance between host immune defense and immune tolerance plays an important role in fungal infectious diseases (29). On the one hand, immune response mechanisms can activate the body’s pro-inflammatory cascade, resulting in resistance to the invasion of pathogenic fungi and the destruction of the pathogen. However, they must suppress the body’s immune response to avoid excessive destruction of normal tissues and limit the expansion of damage to the immune system (30). An increasing number of studies have confirmed that fungal immune tolerance plays an important role in fungal infectious diseases, helping maintain a certain range of immune-inflammatory responses in the host, preventing excessive destruction of the organism, and potentially causing chronic fungal infection, mediating the immune escape of fungi and leading to their persistence in the body (31). The specific mechanisms that maintain immune tolerance and immune resistance in FK are unclear. Fungal keratitis (FK) is a sight-threatening infection often caused by Aspergillus fumigatus, and macrophages play a pivotal role in the host immune response. Previous studies have shown that macrophage polarization contributes significantly to fungal clearance and disease outcome. For example, Honokiol and pseudolaric acid B have been reported to affect macrophage behavior in FK models, and tubular epithelial cell-derived exosomal miRNAs (e.g., miR-19b-3p, miR-374b-5p) have been shown to modulate M1 macrophage activation via SOCS1 targeting in renal inflammation models. However, the role of exosome-derived miRNAs in the macrophage response during fungal keratitis remained unclear. Our study demonstrates that exosomes secreted by A. fumigatus-stimulated human corneal epithelial cells (HCECs) promote M1 macrophage polarization, characterized by elevated expression of iNOS, phosphorylated NF-κB p65, and pro-inflammatory cytokines (e.g., TNF-α, IL-6, MCP-1). Transcriptomic analysis of these exosomes revealed an enrichment of let-7b-5p, which we confirmed as a key regulator of macrophage activation via SOCS1 suppression. The dual-luciferase assay validated SOCS1 as a direct target of let-7b-5p. These findings define a novel exosome/let-7b-5p/SOCS1 axis contributing to M1 polarization in FK, expanding our understanding of innate immune signaling in fungal infections.

Among fungal infectious diseases, aspergillosis is particularly prevalent, and this study focused on the macrophage immune mechanisms associated with *A. fumigatus*-related FK disease. After fungal infection, inflammatory infiltration of tissues and vasodilation of the surrounding tissues usually occur, which leads to the activation and accumulation of a large number of neutrophils, macrophages, dendritic cells, and other immune cells at the site of inflammation. These immune cells can secrete inflammatory factors that mediate inflammatory reactions and trigger an immune response (32). Macrophages are phagocytic and antigen-presenting cells that can engulf fungi. Macrophages are characterized by diversity and plasticity, serving as the first line of the innate immune defense system. They can activate the immune system and thereby trigger the immune response (33). Leonardi et al. demonstrated that inducing macrophage activation may serve as a potential therapeutic strategy for inflammatory bowel disease (34). Gander-Bui et al. reported that the secretion of IL-1Ra by activated macrophages should be viewed as an additional biomarker and a possible target for treatment in cases of severe systemic candidiasis (35). Exosomes secreted by various immune cells such as dendritic cells, neutrophils, and T lymphocytes have been shown to exert regulatory effects on inflammation and host defense. For instance, exosomes derived from γδ T cells were reported to suppress autoimmune uveitis by modulating myeloid cell responses (36). Additionally, neutrophil-derived exosomes play a dual role in inflammation by either promoting or resolving inflammatory processes depending on the activation state (37). These studies highlight the complex interplay between immune cell-derived exosomes and inflammatory signaling pathways, further supporting the significance of our findings in the context of immune modulation during fungal keratitis. Echoing these studies, our findings also revealed that macrophage-associated biological functions were altered, and their secretion of inflammatory factors increased after *A. fumigatus* infection of HCECs, suggesting that the organism’s macrophages begin to function in response to fungal interference. In addition, several researchers have demonstrated the involvement of macrophages in regulating the immune response to pulmonary Cryptococcus infection (38). In conclusion, it appears that macrophages play a crucial role in fungal keratitis.

There are many factors that influence macrophage polarization. In some infectious diseases, exosomes, a type of extracellular vesicle that **is** widely present in the body and secreted by cells, play a key role in cellular communication and may regulate macrophage polarization (39). Teng et al. suggested that miRNAs in exosomes can influence lung inflammation caused by viral infections by affecting macrophages in lung tissue (40). Exosomal miR-192-5p derived from hepatocytes is crucial for activating pro-inflammatory macrophages in advanced non-alcoholic fatty liver disease (NAFLD) by influencing the Rictor/Akt/FoxO1 signaling pathway (39). These studies collectively illustrate that exosomes play an important role in the immune functions of macrophages. One limitation of our study is the lack of direct measurement of exosome particle count. Although we used 10 µg/mL of exosomal protein for macrophage stimulation—consistent with previous studies—a precise correlation between protein concentration and particle number was not established. In addition, we did not perform a full dose-response analysis to assess the graded effects of exosomes on M1 macrophage activation. Future experiments will include nanoparticle quantification and concentration-dependent functional assays to refine our understanding of exosome-mediated immune modulation.

Our findings on exosomal let-7b-5p promoting M1 macrophage activation align with recent studies investigating the role of exosomal miRNAs in macrophage-driven inflammation. Notably, exosomal miR-19b-3p has been demonstrated to enhance M1 macrophage polarization via SOCS1 suppression and NF-κB activation, contributing to inflammatory responses in kidney injury (19). This supports the notion that exosomal miRNAs act as key regulators of macrophage function and are crucial in immune responses to infections. Another limitation of this study is that M2 macrophage markers such as IL-10, Arg-1, or CD206 were not examined. While we observed clear evidence of M1 polarization following exposure to exosomes derived from *A. fumigatus*-treated HCECs, the absence of M2 marker analysis limits the ability to fully exclude alternative polarization outcomes. Future studies will assess both M1 and M2 polarization markers to comprehensively characterize the immunological profile induced by exosomal let-7b-5p. Additionally, research on exosomal miR-374b-5p has shown that it exacerbates renal ischemia/reperfusion injury by inducing M1 macrophage activation through a mechanism involving SOCS1 suppression (20). This aligns with our findings that exosomal let-7b-5p suppresses SOCS1 to promote M1 polarization in fungal keratitis, suggesting that SOCS1 modulation via exosomal miRNAs is a conserved mechanism across multiple inflammatory conditions.

Furthermore, in the context of diabetic kidney disease, extracellular vesicles from albumin-treated tubular epithelial cells were found to induce M1 polarization through miR-199a-5p, which targets Klotho—a critical modulator of inflammation (21). These findings reinforce the broad role of exosome-derived miRNAs as mediators of macrophage-driven inflammation.

While previous studies have focused on kidney-related inflammation, our study expands on this growing body of research by identifying exosomal let-7b-5p as a novel modulator of M1 polarization in fungal keratitis. The observed SOCS1 suppression and NF-κB activation in our study mirror the mechanisms described in kidney injury models, reinforcing the idea that exosome-mediated miRNA transfer serves as a conserved pathway in inflammation-related diseases.

Given the potential role of exosomal miRNAs in immune modulation, our findings suggest that targeting exosomal let-7b-5p could be a promising therapeutic strategy for fungal keratitis and other inflammatory diseases.

In addition, exosomes have the ability to transfer IFN-related miRNAs from macrophages to HBV-infected hepatocytes, demonstrating their antiviral effects on HBV replication and expression (41). Thus, it is clear that exosomes also play a crucial role in fungal infectious diseases. The primary determinants of exosomal function are the bioactive molecules they carry, including proteins, RNAs, and other signaling components. The biological effects of exosomes are primarily determined by their molecular cargo, including miRNAs, proteins, and lipids. To identify which component contributes to macrophage activation, we performed miRNA sequencing on exosomes secreted by A. fumigatus-treated HCECs and identified let-7b-5p as a significantly enriched miRNA candidate. Among the thousands of identified miRNAs, let-7b-5p has been reported to be involved in regulating the course of several human diseases. For example, let-7b-5p can inhibit multiple myeloma by targeting IGF1R and may serve as a target for the treatment and amelioration of multiple myeloma (42). Moreover, let-7b-5p has also been reported to be involved in LncRNA ROR regulation in atherosclerosis (43). Downregulation of the Fas cell surface death receptor (Fas) by let-7b-5p affects the survival of *Mycobacterium tuberculosis* in THP-1 human macrophages (44). Li et al. revealed that exosomes derived from macrophages enhance vascular calcification associated with chronic kidney disease by influencing the let-7b-5p/TGFBR1 pathway in high-phosphate environments (45).

TEC-derived exosomal miR-19b-3p was internalized by macrophages, leading to M1 phenotype polarization through targeting NF-κB/SOCS-1. A dual-luciferase reporter assay confirmed that SOCS-1 was the direct target of miR-19b-3p. Importantly, the pathogenic role of exosomal miR-19b-3p in initiating renal inflammation was revealed by the ability of adoptively transferred purified TEC-derived exosomes to cause tubulointerstitial inflammation in mice, which was reversed by inhibition of miR-19b-3p. Clinically, high levels of miR-19b-3p were found in urinary exosomes and were correlated with the severity of tubulointerstitial inflammation in patients with diabetic nephropathy (21).

In our study, we found that let-7b-5p in exosomes was able to increase the expression level of let-7b-5p in macrophages after being internalized by macrophages. When we transfected and inhibited let-7b-5p in macrophages using a let-7b-5p inhibitor, we were able to reverse the pro-inflammatory effect of exosomes secreted by *A. fumigatus*-stimulated HCECs in macrophages and inhibit the secretion of inflammation-related cytokines. Moreover, the dual-luciferase assay showed that let-7b-5p could target SOCS-1, whereas overexpression or inhibition of let-7b-5p could decrease or increase SOCS-1 expression, respectively, suggesting that let-7b-5p plays a role in regulating SOCS-1.

In this study, we expand on this concept by identifying exosomal let-7b-5p as a novel regulator of M1 macrophage activation in fungal keratitis. The suppression of SOCS1, a well-established inhibitor of cytokine signaling, mirrors the mechanisms observed in renal pathologies, further solidifying the role of exosome-mediated miRNA transfer in macrophage-driven inflammation. These insights not only enhance our understanding of FK immunopathogenesis but also open avenues for potential therapeutic targeting of exosomal miRNA pathways. Although our study provides novel insights into the role of exosomal let-7b-5p in M1 macrophage polarization, one notable limitation is the lack of validation in an animal model. While *in vitro* experiments offer a controlled environment for studying exosomal miRNA-mediated immune modulation, they cannot fully replicate the complex interactions occurring in a live biological system during fungal keratitis progression. Future studies will focus on developing fungal keratitis animal models to evaluate the *in vivo* effects of exosomal let-7b-5p on corneal inflammation, immune cell recruitment, and antifungal defense mechanisms. These investigations will provide deeper insight into the therapeutic potential of targeting exosomal miRNAs in fungal keratitis treatment, advancing their translational application. Furthermore, while our study primarily examines exosomes derived from A. fumigatus-treated human corneal epithelial cells (HCECs), we acknowledge that exosomes from other ocular and immune cells may also play a significant role in fungal keratitis pathogenesis. Exosomes released by macrophages, neutrophils, or dendritic cells in response to fungal infections could contribute to immune modulation, influencing both pro-inflammatory (M1) and anti-inflammatory (M2) macrophage polarization. Similarly, exosomes from corneal fibroblasts or endothelial cells may participate in the host defense response by regulating immune signaling pathways. Future studies should investigate the exosome network in fungal keratitis, exploring how exosomes from different ocular and immune cell sources contribute to macrophage polarization and disease progression. Expanding this research will enhance our understanding of exosome-mediated immune regulation in fungal keratitis, potentially identifying novel therapeutic targets for intervention. Despite these limitations, our findings establish a molecular mechanism linking exosomal let-7b-5p to M1 macrophage activation via SOCS1 suppression, providing a strong foundation for future research. Given the increasing interest in exosome-based therapies, this study serves as a preliminary step toward elucidating the broader exosome crosstalk in fungal keratitis, paving the way for future translational applications. Interestingly, previous research has demonstrated a contrasting role of let-7 family miRNAs in immune polarization. For example, let-7-enriched exosomes derived from T. pisiformis cysticercus have been shown to suppress M1 polarization and enhance M2 phenotype (PMID: 34209741). This divergence may reflect the context-dependent effects of miRNAs, particularly influenced by the type of pathogen (fungal vs. parasitic), the cellular microenvironment, and the specific molecular targets engaged. In our study, exosomal let-7b-5p promotes M1 polarization primarily via SOCS-1 inhibition, enhancing NF-κB signaling. Such differences underscore the complexity of miRNA-mediated immune regulation and highlight the importance of disease-specific studies in interpreting miRNA function.

## Future clinical applications and considerations

Our findings not only provide new insights into the role of exosomal let-7b-5p in fungal keratitis but also open avenues for its potential clinical application as a diagnostic and therapeutic target. As a diagnostic biomarker, exosomal let-7b-5p could be detected in accessible biological fluids, such as tear fluid or serum, using sensitive assays like quantitative PCR or digital droplet PCR. However, translating these methods into clinical practice will require standardization of exosome isolation and quantification protocols to ensure reproducibility across different laboratories. On the therapeutic front, strategies to modulate the let-7b-5p regulated pathway—either by employing specific inhibitors or mimics—could offer novel treatments for fungal keratitis. Yet, challenges remain in ensuring the targeted delivery of these therapeutic agents, minimizing off-target effects, and managing potential immune responses. Additionally, ethical considerations must be addressed, including obtaining informed consent for the use of patient-derived samples, safeguarding patient data, and adhering to regulatory guidelines during clinical trials. Comprehensive preclinical studies and well-designed clinical trials will be essential to overcome these technical and ethical hurdles before clinical implementation can be realized.

## Conclusion

In the present study, HCECs were stimulated with *A. fumigatus*, and the secreted exosomes were harvested. These exosomes were shown to induce macrophage M1-type polarization. Subsequently, let-7b-5p, enriched in exosomes, was identified as a key factor in inducing macrophage M1-type polarization. By altering the expression of let-7b-5p and using dual-luciferase assays, we confirmed that let-7b-5p, by targeting SOCS-1, regulates macrophage polarization. This study provides new insights and targets for the treatment and diagnosis of FK. While this study provides strong *in vitro* evidence for the role of exosomal let-7b-5p in M1 macrophage polarization, the absence of animal model validation remains a limitation. Future research will focus on developing fungal keratitis animal models to further investigate the *in vivo* effects of exosomal let-7b-5p on corneal immunity and antifungal responses, ensuring a more comprehensive understanding of its therapeutic potential. Additionally, further studies should explore the exosomal network in fungal keratitis by examining exosomes from other ocular and immune cells, such as macrophages, neutrophils, and dendritic cells. Investigating how exosomes from diverse cellular sources contribute to macrophage polarization and immune modulation will provide deeper insights into exosome-mediated signaling in fungal keratitis and may uncover novel therapeutic targets for improved disease management.

## Limitations and future perspectives

This study sheds light on a novel mechanism by which exosomal let-7b-5p, derived from A. fumigatus-stimulated human corneal epithelial cells, promotes M1 macrophage activation through SOCS1 suppression. While our findings represent a significant step forward in understanding the immune modulation of fungal keratitis, they also open up several important directions for continued exploration. Our use of *in vitro* models enabled precise dissection of the molecular interactions underlying macrophage polarization; however, the complexity of immune regulation in fungal keratitis cannot be fully captured outside a living system. Therefore, validating these results in an *in vivo* A. fumigatus keratitis model is a natural next step. Such studies will provide a more comprehensive view of how exosomal let-7b-5p influences corneal inflammation, immune cell recruitment, and host defense mechanisms within a physiological context. Additionally, while our current work focused on M1 macrophage markers, the assessment of M2 polarization was beyond its scope. Given the dynamic plasticity of macrophages, a balanced evaluation of both M1 and M2 markers in future experiments will help to fully characterize the immunological phenotype shaped by exosomal signaling.

In terms of exosome quantification, we utilized protein concentration as a practical and widely accepted proxy. Nonetheless, integrating nanoparticle tracking analysis and performing dose-response studies in future work will allow for a more nuanced understanding of exosome-mediated immune activation and ensure consistency across experimental conditions. Finally, it is important to recognize that the immune landscape in fungal keratitis is shaped by diverse cellular players. While our study centered on epithelial-derived exosomes, immune and stromal cells such as macrophages, neutrophils, and fibroblasts are also likely contributors to exosome-mediated signaling. Exploring the interactions between these exosomal sources will deepen our understanding of how innate immunity is orchestrated at the ocular surface. Taken together, these future directions are not merely acknowledgments of current boundaries but opportunities to build upon a solid mechanistic foundation. By extending this line of inquiry into *in vivo* models and broader exosomal networks, we hope to contribute to the development of targeted immunotherapies for fungal keratitis and related inflammatory diseases. Our findings provide a timely entry point into this emerging field, supporting the continued integration of exosome biology into ocular immunopathology research.

## Data Availability

The original contributions presented in the study are publicly available. The data supporting the findings of this study, including raw images, figures, and supplementary documents, have been deposited in Figshare and are accessible via the following DOI: https://doi.org/10.6084/m9.figshare.29194547.
