# Correction: Exosomal let-7b-5p derived from Aspergillus fumigatus-treated human corneal epithelial cells promotes M1 macrophage activation via targeting SOCS-1

**DOI:** 10.3389/fimmu.2025.1643508

**Published:** 2025-08-05

**Authors:** Xiaoming Yu, Xinyi Wu

**Affiliations:** 1 Department of Ophthalmology, Qilu Hospital of Shandong University, No.107, Wenhua Xilu, Jinan, Shandong, China; 2 Department of Ophthalmology, Shandong Provincial Third Hospital, Shandong University, Jinan, Shandong, China

**Keywords:** fungal keratitis, exosomes, let-7b-5p, SOCS-1, M1 macrophage activation

There was a mistake in **Figure 1** as published. **Figure 1A**, title changed to HEX^PBS^ (PBS superscript); **Figure 1C**, first target protein labelled as Calnexin. The corrected **Figure 1** appears below.

**Figure 1:**
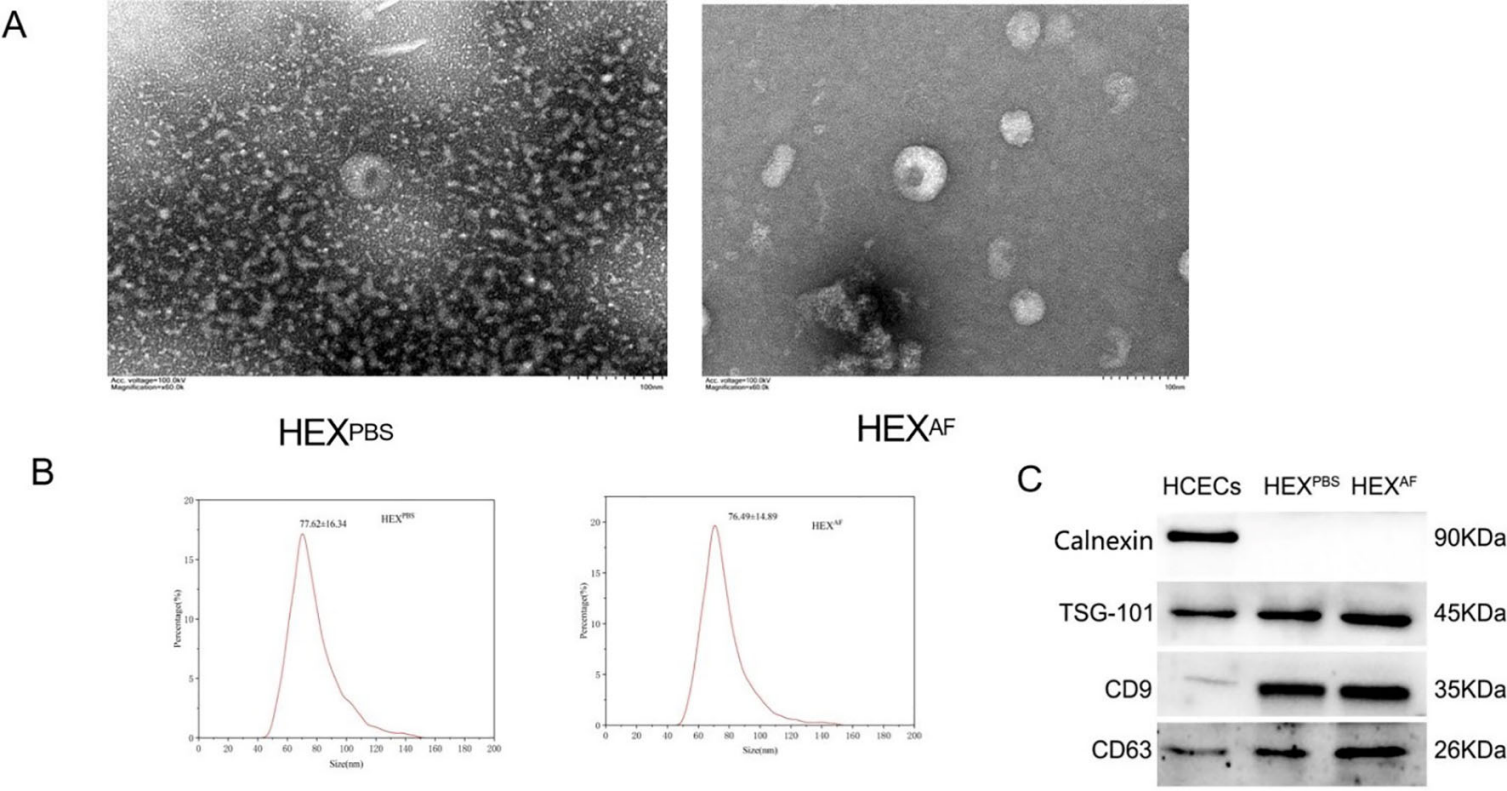
Identification of exosomes. **(A)** Detection of exosome morphology by transmission electron microscopy (200×); **(B)** Nanoflow technology to detect exosome size; **(C)** Exosome marker identification. HEX^PBS^, exosomes secreted by PBS-treated HCECs, HEX^AF^, exosomes secreted by *A. fumigatus*-treated HCECs.

The original version of this article has been updated.

